# Investigation on the Micro-Segregation Behaviors of a High-Mn Austenitic Cryogenic Steel Continuous Casting Slab Through Thermodynamic Calculations and Homogenization Experiments

**DOI:** 10.3390/ma19102109

**Published:** 2026-05-17

**Authors:** Tao Liu, Yu Du, Chao Sun, Xiuhua Gao, Hongyan Wu, Linheng Chen, Linxiu Du

**Affiliations:** 1 State Key Laboratory of Digital Steel, Northeastern University, Shenyang 110819, China; liut12@mails.neu.edu.cn (T.L.); gaoxh@ral.neu.edu.cn (X.G.); wuhy@ral.neu.edu.cn (H.W.); dulx@ral.neu.edu.cn (L.D.); 2 Jiangsu Key Laboratory for Premium Steel Material, Nanjing Iron & Steel Co., Ltd., Nanjing 210035, China; duyu_2016@foxmail.com (Y.D.); chaos_83@163.com (C.S.)

**Keywords:** high-Mn steel, austenite, cryogenic steel, continuous casting slab, micro-segregation

## Abstract

This study systematically investigated the mechanism of micro-segregation reduction in a high-Mn austenitic cryogenic steel continuous casting slab using thermodynamic calculations and homogenization experiments. The high-Mn austenitic cryogenic steel continuous casting slab exhibits obvious non-equilibrium solidification characteristics, with severe interdendritic segregation of C and Mn. The solidus temperatures of equilibrium, Scheil, and Scheil–Back solidification are 1324 °C, 953 °C, and 1272 °C, respectively. According to thermodynamic calculations, there is only a slight decrease in the highest segregation C content when the homogenization temperature is 900 °C. When the specimens were homogenized at 1000 °C, the segregation of C and Mn was significantly alleviated, and the segregation degree further decreased when the homogenization temperature was 1100 °C. Two feasible and industrially applicable strategies for alleviating the micro-segregation of a high-Mn steel continuous casting slab are proposed. First, reduce the cooling intensity of the secondary cooling stage during continuous casting, slow down the cooling rate around 1000 °C, and promote the limited diffusion of solute elements to reduce initial segregation. Second, introduce a holding stage at around 1000 °C during slab reheating prior to hot rolling, eliminating residual segregation and stabilizing the local solidus temperature above 1200 °C.

## 1. Introduction

Nowadays, 9Ni steel and austenitic stainless steel are widely used for the storage and transportation equipment of liquefied natural gas (LNG) and liquid hydrogen (LH2) [1,2,3]. However, these steels have higher costs due to the addition of a large amount of alloys. Furthermore, high-Mn austenitic cryogenic steels are becoming a core candidate material for the storage and transportation equipment of LNG and LH2, owing to their exceptional cryogenic toughness due to their excellent austenite stability under extreme low temperatures [4,5,6]. Different from conventional low-alloy steels, high-Mn steel exhibits unique non-equilibrium solidification behavior during continuous casting because of its ultra-high-Mn and moderate C contents. The solidification of high-Mn steel has a wide temperature range, which leads to significant solute enrichment in the interdendritic region during continuous casting [7,8,9,10]. Micro-segregation not only induces carbide and banded structures in continuous casting slabs, but also reduces the local melting point in interdendritic areas, causing surface liquefaction and cracking during hot rolling. These defects compromise the cryogenic mechanical properties and limit the engineering application of high-Mn steel.

Therefore, it is necessary to reduce the micro-segregation of a high-Mn steel continuous casting slab before hot rolling, and a commonly used method to reduce micro-segregation is homogenization heat treatment. As for high-Mn steel, excessive temperature can easily cause local melting due to micro-segregation, while an excessively low temperature requires very long times. At present, most research is focused on the segregation tendencies and their impact on the mechanical properties of high-Mn steel. Wietbrock et al. discovered that the maximum micro-segregation increments of Mn in Fe-22Mn-0.30C high-Mn steel ingots reached 7.0 wt% [11]. Lan et al. employed EPMA to analyze the Mn and C distribution between dendritic structures in Fe-22Mn-0.7C high-Mn steels, revealing significant positive segregation phenomena between dendrites. These micro-segregations were found to compromise microstructural uniformity, thereby affecting high-temperature mechanical performance [12]. Lan et al. further investigated the interrelationships of solute element distribution at the microscale, demonstrating a pronounced positive correlation between Mn and C segregation ratios at dendritic dimensions [13]. Hecht et al. successfully reduced the Mn micro-segregation increment from 9.0 wt% to 4.8 wt% in Fe-21Mn-0.3C ingots through electroslag remelting [14]. However, there is still little research on the homogenization process of non-equilibrium solidification micro-segregation microstructures in high-Mn steel.

In this study, the micro-segregation reduction mechanism of a high-Mn austenitic cryogenic steel continuous casting slab was systematically analyzed using Thermo-Calc thermodynamic calculations and homogenization experiments. The degree of micro-segregation that caused defects was quantitatively determined. This research aims to provide reliable theoretical support and practical technical parameters for controlling micro-segregation defects and promoting the application of high-Mn steel.

## 2. Experimental Materials and Methods

The studied steel was a 260 mm-thick high-Mn austenitic cryogenic steel continuous casting slab produced by Nanjing Iron & Steel Co., Ltd., Nanjing, China, and the chemical composition of the steel is listed in Table 1. In order to investigate the effects of homogenization temperature and time on the micro-segregation of high-Mn steel, the alloy distribution during solidification and homogenization processes were calculated using Thermo-Calc software (2023b) coupled with the TCFE12 and MOBFE7 steel thermodynamic database for simulation calculations. Three solidification models were adopted for comparative analysis, which are equilibrium, Scheil, and Scheil–Back. The solidification calculation ends at a liquid phase fraction of 0.01%. Because Scheil has the most severe micro-segregation, the element distribution after Scheil solidification was used for diffusion calculations. The homogenization process was simulated through the following parameters. Half of the spacing between secondary dendrite arms was set to 100 μm, and the homogenization temperatures were 900 °C, 1000 °C, and 1100 °C and the homogenization times were 1, 2, and 6 h, respectively. The micro-segregation degrees of different homogenization process were obtained, and the melting temperature can also be calculated.

The homogenization experiment specimens were sampled on the upper surface at one quarter of the width. Based on the results of thermodynamic calculations, homogenization experiments were conducted at 900 °C, 1000 °C, and 1100 °C for 2 h. After homogenization experiments, the element distributions were analyzed using field emission electron probe microanalysis (EPMA, JXA-8530F JEOL Ltd., Akishima, Japan). A protective atmosphere heating experiment was conducted on the specimens before and after homogenization at 900 °C, 1000 °C, and 1100 °C for 2 h with argon gas. The specimens were cut near the top of the slab and the dimensions of the specimens were 20 mm thick, 30 mm width, and 50 mm length. The specimens required grinding and polishing before the protective atmosphere heating experiment. They were heated from room temperature to 1200 °C and isothermal for 1 h at a heating rate of 5 °C/s, and then cooled to room temperature with argon gas. Finally, the grain boundary was observed using field emission scanning electron microscope (SEM, ULTRA 55, Carl Zeiss AG, Oberkochen, Germany).

## 3. Thermodynamic Calculation Results

**Figure 1:**
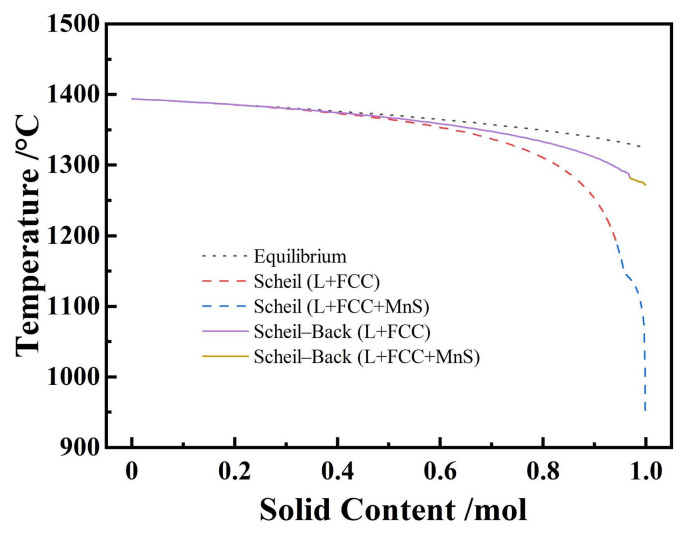
Solidification path diagram of experimental steel calculated using Thermo-Calc.

Due to its high alloy content, high-Mn steel will undergo segregation during the solidification process, which will result in a significantly lower local melting temperature than plain carbon steel. Figure 1 shows the solidification path diagram of experimental steel, and the liquidus temperature and the solidus temperature of different solidification paths are listed in Table 2. The solidus temperatures of equilibrium, Scheil, and Scheil–Back solidification are 1324 °C, 953 °C, and 1272 °C. The actual solidus temperature should be between Scheil and Scheil–Back solidification; it can be reasonably inferred that the local solidus temperature is between 1100 °C and 1200 °C, considering that the actual local segregation is not as severe as theoretically calculated.

**Figure 2:**
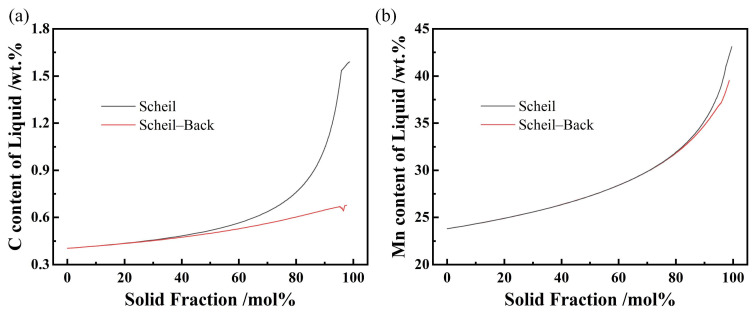
C and Mn content of liquid during solidification. (**a**) C content, (**b**) Mn content.

The low solidus temperature of the slab is mainly due to the local segregation of alloy elements, which can be considered to be mainly caused by C and Mn in the experimental steel. In Figure 2, it can be seen that the largest C content in the interdendritic region is about 1.6% and the largest Mn content in the interdendritic region is about 44% in Scheil mode. However, the highest C and Mn content is 0.6% and 38% in Scheil–Back mode, respectively. It can be clearly seen that the segregation of C is much greater than that of Mn, because C has a smaller atomic weight and a higher diffusion coefficient than Mn. Severe dendritic micro-segregation is the core influence causing local melting point depression in slabs, and Mn- and C-enriched regions exhibit significantly lower melting points than the matrix. When heated to conventional slab reheating temperatures (1200 °C), local liquefaction readily occurs, resulting in surface cracks, spalling, and other defects that severely compromise slab surface quality and processability.

Specifically, the interdendritic regions with high C and Mn concentrations form low-melting-point eutectic phases. These phases melt preferentially during heating, causing the loss of dendritic bonding forces and subsequent hot cracking [15,16,17]. Furthermore, the heterogeneous alloy distribution results in non-uniform austenite stability across the slab. Regions with a high Mn content exhibit enhanced austenite stability, whereas dendrite cores with a low Mn content undergo martensitic transformation at cryogenic temperatures. This transformation exacerbates mechanical property inhomogeneity after hot rolling, particularly compromising cryogenic toughness—a critical parameter for LNG and LH2 storage equipment. The disparity in segregation severity between C and Mn correlates directly with their diffusion characteristics. Carbon, with its smaller atomic radius, diffuses rapidly even at moderate temperatures. Conversely, Mn—characterized by a larger atomic radius and higher diffusion activation energy—exhibits sluggish diffusion kinetics, resulting in lower segregation intensity compared to C [18,19,20,21].

**Figure 3:**
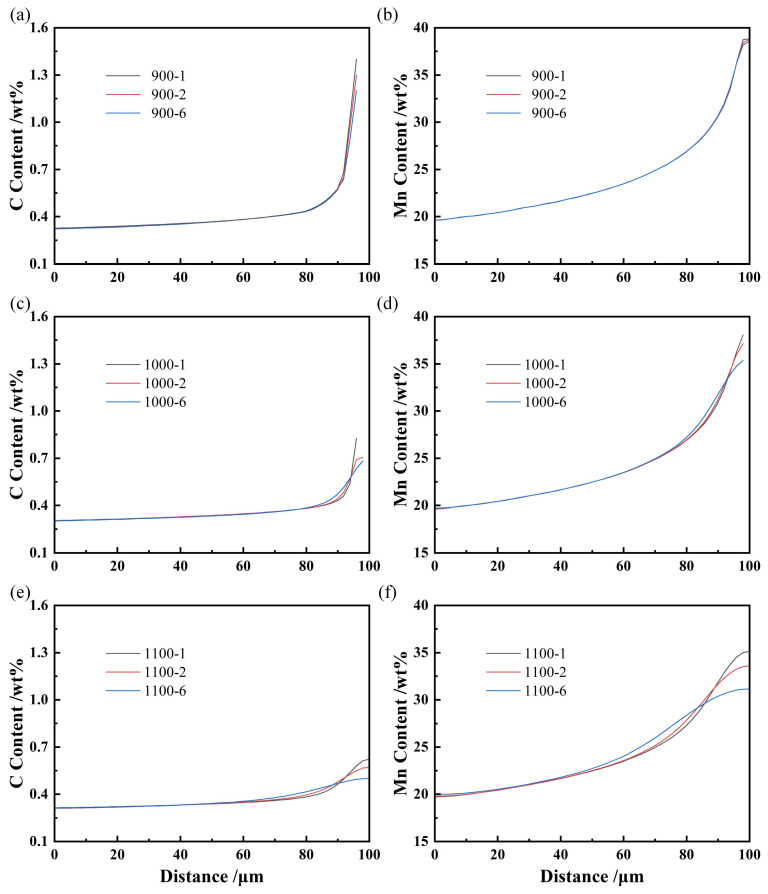
The element distributions after different homogenization conditions. (**a**) C-900 °C, (**b**) Mn-900 °C, (**c**) C-1000 °C, (**d**) Mn-1000 °C, (**e**) C-1100 °C, (**f**) Mn-1100 °C.

Since there is severe element segregation in the slab, it is necessary to treat the billet to reduce segregation, and homogenization heat treatment is a common method to reduce segregation. For high-Mn steel, the homogenization temperature needs to be controlled within a reasonable range. Excessive temperatures can cause grain boundary damage, while insufficient temperatures cannot achieve sufficient homogenization. Therefore, when using the Thermo-Calc Scheil solidification and diffusion module for calculations, homogenization at 900 °C, 1000 °C, and 1100 °C for 1 h, 2 h, and 6 h was selected. The element distributions after different homogenization conditions are shown in Figure 3, and the local solidus temperatures calculated from the element distribution in Figure 3 are listed in Table 3. When the homogenization temperature is 900 °C, there is only a slight decrease in the highest segregation C content, while the highest segregation Mn content remains almost unchanged for the duration of homogenization. This is because C can still diffuse slightly, while Mn hardly diffuses at 900 °C. When the homogenization temperature is 1000 °C, there is a significant decrease in the content of C and Mn, and there is also a noticeable decrease in the segregation with the extension of homogenization time. The C content is around 0.70% and the Mn content is around 37%. Furthermore, the highest segregation content further decreases when the homogenization temperature is 1100 °C, the C content is around 0.55% and the Mn content is around 33%. Furthermore, the local solidus temperature after different homogenization conditions was calculated based on the element distributions after different homogenization conditions. It can be found that homogenization at 1000 °C and 1100 °C will cause the local solidus temperature to exceed 1200 °C. Taking into account all factors, it can be considered that homogenization at 1000 °C for 2 h is a more suitable parameter.

## 4. Experimental Results and Discussion

Through the previous thermodynamic calculations, it was found that homogenization treatment can reduce micro-segregation, thereby lowering the local melting temperature. The calculation results show that the melting temperature can exceed 1200 °C when homogenized for 1, 2, and 6 h at 1000 °C, but the magnitude of the excess is relatively small. However, the actual situation may differ slightly from the calculated results, and experimental verification is needed in order to verify this rule. In addition, the time spent in the high temperature range is mainly around 2 h in actual production. Therefore, a protective atmosphere heating experiment (1200 °C for 1 h) was conducted on the specimens before and after homogenization at 900 °C, 1000 °C, and 1100 °C for 2 h.

**Figure 4:**
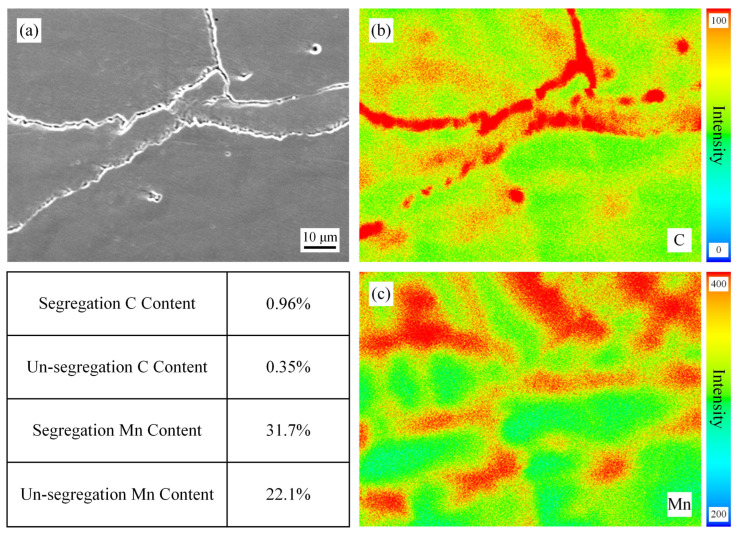
EPMA results of the specimens without homogenization. (**a**) SEM, (**b**) Distribution of C, (**c**) Distribution of Mn.

**Figure 5:**
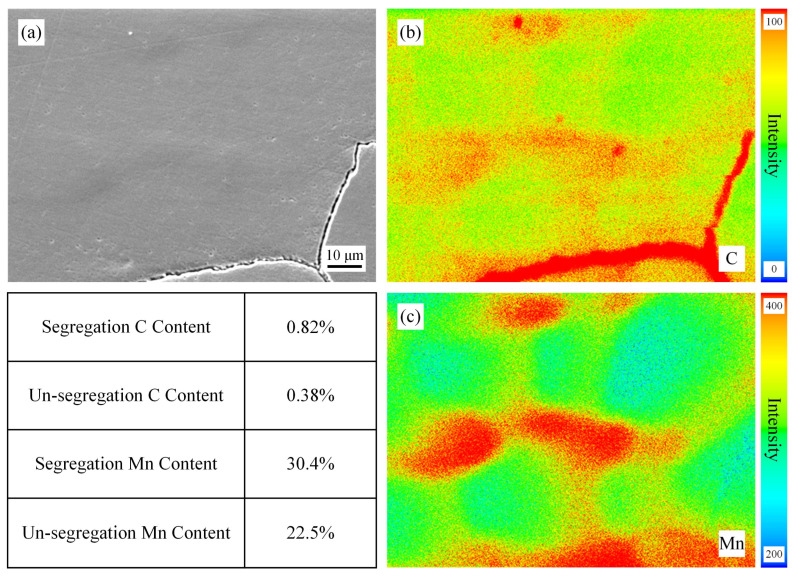
EPMA results of the specimens after homogenization at 900 °C for 2 h. (**a**) SEM, (**b**) Distribution of C, (**c**) Distribution of Mn.

**Figure 6:**
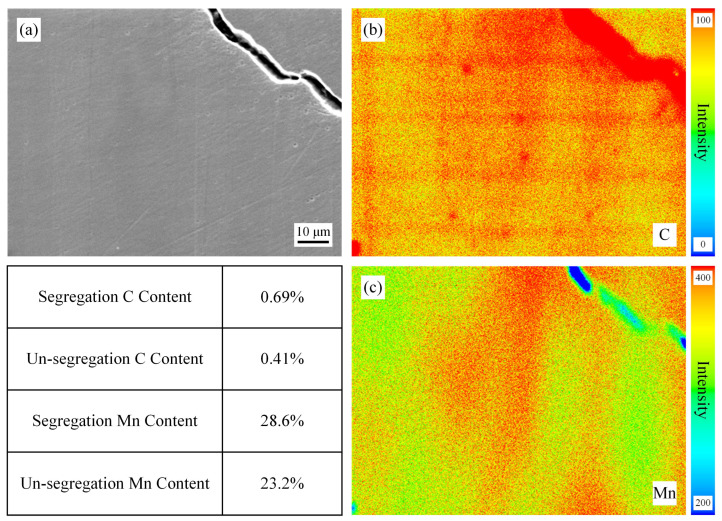
EPMA results of the specimens after homogenization at 1000 °C for 2 h. (**a**) SEM, (**b**) Distribution of C, (**c**) Distribution of Mn.

**Figure 7:**
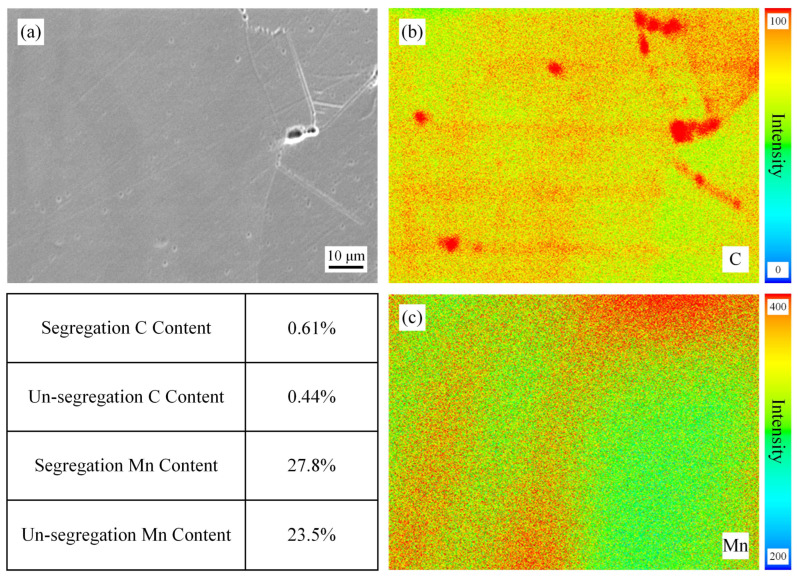
EPMA results of the specimens after homogenization at 1100 °C for 2 h. (**a**) SEM, (**b**) Distribution of C, (**c**) Distribution of Mn.

Figure 4 shows the EPMA maps of the surface position of the slab without homogenization treatment. For the accuracy of this EPMA experiment, these specimens were tested at the same time and calibrated with standard samples before testing. Despite these efforts, we can only make semi-quantitative comparisons based on the results. From the figure, it can be seen that the segregation of C and Mn is very obvious, with the highest C and Mn contents at the segregation site being 0.96% and 31.7%, respectively. The dendrite cores are light-colored (low solute content), while the interdendritic regions are dark-colored (high solute content), showing a typical dendritic segregation morphology. Figure 5, Figure 6 and Figure 7 show the EPMA maps of the surface position of the slab after homogenization at 900 °C, 1000 °C, and 1100 °C for 2 h. As can be seen in these figures, the segregation weakens with the increase in homogenization temperature, which is consistent with the thermodynamic calculation results. Yet the degree of improvement in segregation measured in the experiment is lower than the results of thermodynamic calculations, but the pattern is the same. An obvious phenomenon can also be observed that when the homogenization temperature is increased from 900 °C to 1000 °C: the degree of reduction in segregation is more pronounced, indicating that the diffusion of C and Mn will significantly accelerate above 1000 °C. This is because the diffusion coefficient of solute elements increases exponentially with temperature according to the Arrhenius equation, and 1000 °C is close to the temperature for Mn diffusion in the austenite matrix, leading to a sharp increase in diffusion rate [22]. The gap between the experimental and calculation results is mainly due to the idealized assumptions in the calculation, while in actual experiments, the surface of the slab is prone to slight oxidation during homogenization, resulting in a small amount of C loss, and the temperature distribution in the 260 mm thick slab is not completely uniform, leading to insufficient diffusion in local regions. In addition, the EPMA characterization results also show that the segregation of C is more easily alleviated than that of Mn, which is consistent with the thermodynamic calculation results, further confirming that the diffusion rate of C is significantly higher than that of Mn.

**Figure 8:**
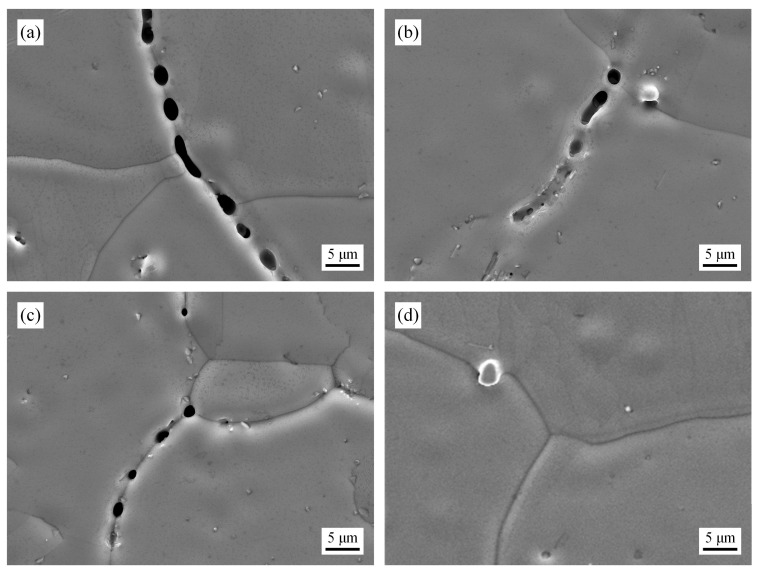
Protective atmosphere heating results of the specimens. (**a**) Unhomogenized, (**b**) 900 °C, (**c**) 1000 °C, (**d**) 1100 °C.

Figure 8 shows SEM photos of the specimens after the protective atmosphere heating experiment. It can be seen that as the homogenized temperature increases, the melting area gradually decreases and completely disappears at 1200 °C. Even so, the melting area of the sample homogenized at 900 °C is still smaller than that of the original slab, indicating that even low-temperature homogenization can slightly alleviate segregation and reduce local melting. In thermodynamic calculations, melting should not occur at 1000 °C, but in reality, there is still a small amount of melting at 1000 °C, indicating that the actual homogenization treatment is weaker than the calculation. The optimal homogenization temperature should be between 1000 °C and 1100 °C. The residual melting at 1000 °C in the experiment is mainly due to the incomplete diffusion of Mn atoms in the actual homogenization process: although the homogenization at 1000 °C can significantly reduce the segregation of C and Mn, there are still local small regions with a high solute content, whose solidus temperature is lower than 1200 °C, leading to local liquefaction during reheating. In contrast, the specimen homogenized at 1100 °C has no melting phenomenon, but grain boundary melting may occur during the homogenization process at 1100 °C, based on the results of thermodynamic calculations of the solidus temperature. Therefore, comprehensively considering the segregation elimination effect and grain boundary damage, the homogenization temperature should be controlled between 1000 °C and 1100 °C.

Through thermodynamic calculations and EPMA characterization, it can be recognized that element segregation is inevitable in the continuous casting slab of high-Mn steel due to its wide solidification temperature range and unique solute diffusion characteristics. Specifically, the high diffusion activation energy of Mn atoms and the significant difference in diffusion rates between C (~10^−12^ m^2^/s at 1000 °C) and Mn (~10^−14^ m^2^/s at 1000 °C) [23]. This inherent segregation characteristic is further exacerbated by the industrial continuous casting process, where the rapid cooling rate (especially in the secondary cooling stage) limits the time for solute back-diffusion, leading to the retention of severe interdendritic enrichment of C and Mn [24,25]. However, this study provides some new ideas and technical strategies for alleviating element segregation in continuous casting slabs of high-Mn steel. The first method is to slow down the cooling rate at around 1000 °C during the continuous casting process, that is, to reduce the cooling intensity of the secondary cooling stage. This is because 1000 °C is close to the temperature for the diffusion of Mn atoms in the austenite matrix—slowing down the cooling rate in this interval can effectively extend the residence time of the slab in the high-temperature zone, providing sufficient kinetic conditions for the limited diffusion of C and Mn atoms, thereby spreading the segregation formed during the solidification process of the continuous casting slab and reducing the local segregation degree. Specifically, during the secondary cooling stage of continuous casting, the cooling water flow in the segment corresponding to the slab surface temperature of around 1000 °C can be reduced, which can prolong the residence time of the slab at this temperature. This extension promotes the diffusion of C and Mn atoms from the interdendritic regions (high solute concentration) to the dendrite cores (low solute concentration), reducing the concentration gradient between dendrite cores and interdendritic regions compared with the conventional cooling process, and thus effectively alleviating the formation of severe segregation. Importantly, this method does not require additional equipment investment and can be realized by adjusting the water distribution of the existing continuous casting secondary cooling system, which has high industrial applicability and economic benefits. It should be noted that the cooling rate cannot be excessively reduced, otherwise it will lead to the growth of austenite grains, which will damage the mechanical properties of the slab.

The second method is to maintain insulation at around 1000 °C during the rolling heating process, which can further reduce local residual segregation and stably increase the local melting temperature, ensuring that no melting cracks occur when the slab is heated to the conventional reheating temperature (1200 °C) and hot rolled. This strategy is a supplementary measure to the homogenization heat treatment, aiming to solve the problem of incomplete segregation elimination caused by an uneven temperature field and insufficient holding time in the actual homogenization process. Specifically, during the slab reheating process before hot rolling, a holding stage at 1000 °C for 2 h is added on the basis of the conventional reheating system (heating to 1200 °C for 5 °C/min). This holding stage can further promote the diffusion of solute elements, especially Mn atoms, which have low diffusion rates. The EPMA characterization results show that after adding this holding stage, the residual Mn segregation coefficient decreased, and the local solidus temperature could be stably maintained above 1200 °C. This combined heat treatment strategy (homogenization + reheating holding) can effectively balance the segregation elimination effect and production efficiency, and the homogenization treatment at 1000 °C for 2 h lays the foundation for segregation reduction, and the reheating holding stage further eliminates residual segregation, avoiding the defects (such as local melting and grain growth) caused by insufficient homogenization or excessive heating. Moreover, this strategy only needs to adjust the heating curve of the existing reheating furnace, without adding new equipment, which is an easy step to popularize in industrial production.

In addition, the two methods can be used in combination to achieve a synergistic segregation control effect, which is more in line with the actual production needs of high-Mn steel continuous casting slabs. The specific operation process is as follows: First, optimize the secondary cooling process of continuous casting and reduce the cooling intensity in the 950 °C~1050 °C interval to reduce the initial segregation of the slab. Then, perform homogenization treatment at 1000 °C to further alleviate segregation; finally, add a holding stage at 1000 °C during the reheating process before hot rolling to eliminate residual segregation. The combined application of the two methods can reduce the segregation coefficient of Mn and the local solidus temperature can be stably higher than 1200 °C, which completely avoids the occurrence of local liquefaction and hot cracking during reheating and hot rolling. This combined strategy not only improves the quality of the slab, but also reduces the production cost, providing a feasible and economic technical path for the large-scale industrial application of high-Mn austenitic cryogenic steel.

## 5. Conclusions

This study systematically investigated the micro-segregation reduction mechanism of a high-Mn austenitic cryogenic steel continuous casting slab using Thermo-Calc thermodynamic calculations and homogenization simulation experiments. The detailed conclusions are drawn as follows:

(1). The high-Mn austenitic cryogenic steel continuous casting slab exhibits obvious non-equilibrium solidification characteristics, with severe interdendritic segregation of C and Mn. The solidus temperatures of equilibrium, Scheil, and Scheil–Back solidification are 1324 °C, 953 °C, and 1272 °C, respectively. The maximum C and Mn contents in the interdendritic region reach 0.6% and 38% in Scheil–Back mode.
(2). Based on thermodynamic calculations, there is only a slight decrease in the highest segregation C content, when the homogenization temperature is 900 °C. Meanwhile with homogenization at 1000 °C, the segregation of C and Mn is significantly alleviated, and the segregation degree further decreases when the homogenization temperature is 1100 °C. Considering the segregation elimination effect and the solidus temperature, homogenization at 1000 °C is the optimal parameter.
(3). Micro-segregation decreases with the increase in homogenization temperature and time, but the actual segregation improvement degree is lower than the thermodynamic calculation results. Protective heating experiments show that there is still a small amount of local melting in the specimen homogenized at 1000 °C, indicating that the optimal homogenization temperature should be between 1000 °C and 1100 °C.
(4). Two feasible and industrially applicable strategies for alleviating the micro-segregation of high-Mn steel continuous casting slab are proposed. First, reduce the cooling intensity of the secondary cooling stage during continuous casting and slow down the cooling rate around 1000 °C to reduce initial segregation. Second, add a holding stage at around 1000 °C before hot rolling to further eliminate residual segregation.

## Figures and Tables

**Table 1:** Chemical composition of the experimental steel (wt.%).

C	Si	Mn	Cr	P	S	Fe
0.42	0.18	24.0	3.5	0.01	0.001	Bal.

**Table 2:** The liquidus and solidus temperatures of different solidification paths (°C).

	Liquidus Temperature	Solidus Temperature
Equilibrium	1394	1324
Scheil	1394	953
Scheil–Back	1394	1272

**Table 3:** The local solidus temperature after different homogenization temperatures and times (°C).

	1 h	2 h	6 h
900 °C	1094	1109	1115
1000 °C	1206	1229	1246
1100 °C	1249	1263	1287

## Data Availability

The original contributions presented in this study are included in the article. Further inquiries can be directed to the corresponding author.
